# Jiedu-Shengji Ointment promotes diabetic wound repair by inhibiting the NLRP3/Caspase-1/GSDMD signaling pathway and regulating macrophage function

**DOI:** 10.3389/fimmu.2026.1865157

**Published:** 2026-07-06

**Authors:** Letian Guo, Xi Zhang, Yarong Ding, Shuangxi Yang, Tingting Wang, Li Chen, Xinling Huang, Zhongzhi Zhou

**Affiliations:** 1 College of Integrated Traditional Chinese and Western Medicine, Hunan University of Chinese Medicine, Changsha, Hunan, China; 2 Department of Burn Plastic Surgery, The First Hospital of Hunan University of Chinese Medicine, Changsha, Hunan, China; 3 Hunan Provincial Key Laboratory of Vascular Biology and Translational Medicine, Changsha, Hunan, China; 4 College of Traditional Chinese Medicine, Hunan University of Chinese Medicine, Changsha, Hunan, China; 5 College of Medical, Hunan University of Chinese Medicine, Changsha, Hunan, China

**Keywords:** cell pyroptosis, diabetic wound repair, inflammatory regulation, Jiedu-Shengji Ointment, macrophage polarization, NLRP3 inflammasome

## Abstract

**Introduction:**

Diabetic ulcers are one of the most common clinical complications of diabetes. These ulcers are characterized by poor healing and high recurrence rates, which lead to heavy medical burdens and greatly elevate the risk of limb amputation. They have therefore become a critical challenge for clinical tissue repair. Jiedu-Shengji Ointment (JDSJG) is a topical preparation composed of scorpio, scolopendra, frankincense and myrrh. Existing studies have verified its efficacy in promoting diabetic wound healing. Nevertheless, the specific molecular mechanisms responsible for its wound-healing activity remain poorly elucidated. This study aimed to preliminarily explore the molecular mechanisms by which JDSJG facilitates diabetic wound repair.

**Methods:**

We established a mouse model of diabetic skin wounds using streptozotocin (STZ) induction combined with full-thickness skin puncture. We then detected and evaluated multiple indicators of wound tissue, including tissue microstructure and repair status, skin macrophage polarization, the mRNA and protein levels of inflammatory factors, as well as the key gene and protein expression of the NLRP3/Caspase-1/GSDMD signaling pathway. In the *in vitro* experiment, we induced pyroptosis in RAW264.7 cells via lipopolysaccharide (LPS) stimulation. We further observed cellular morphological changes, detected cell pyroptosis and macrophage polarization, and measured the expression of inflammatory factors and key molecules in the NLRP3/Caspase-1/GSDMD pathway. Finally, we adopted LC-MS/MS and GO and KEGG enrichment analyses to clarify the material basis and potential molecular mechanisms of JDSJG.

**Results and discussion:**

In a mouse model of diabetic wounds, treatment with JDSJG effectively alleviated the inflammatory response and significantly promoted the healing process of diabetic wounds in mice. *In vitro* and *in vivo* experiments confirmed that JDSJG downregulated the expression of inflammatory cytokines and inhibited the activation of the NLRP3/Caspase-1/GSDMD pathway. Additionally, flow cytometry results indicated that JDSJG can induce the polarization of macrophages toward the anti-inflammatory M2 phenotype. LC-MS/MS and network pharmacology analyses confirmed that the primary ingredients of JDSJG—scorpio and scolopendra—are associated with pyroptosis. Overall, this study established the therapeutic efficacy of JDSJG in promoting diabetic wound repair and highlighted its potential in regulating wound inflammation and macrophage polarization.

## 1 Introduction

Diabetic wounds are a common and serious complication of diabetes. These wounds often fail to heal, resulting in high medical costs and significantly increasing the risk of amputation. Diabetic wounds have a high incidence. it is estimated that one diabetic patient undergoes amputation due to a foot ulcer every 20 seconds worldwide (1, 2). Although therapeutic approaches such as negative pressure wound therapy, cellular growth factors, and novel dressings have continued to advance in recent years, current diabetic wound treatments still fail to fully meet clinical needs, which highlights the urgent demand for more effective therapeutic strategies.

One of the core pathological features underlying the difficulty in healing diabetic wounds is the persistent local inflammatory response at the wound site (3). Normal wound healing involves four sequential phases: hemostasis, inflammation, proliferation, and remodeling. Timely initiation and resolution of the inflammatory phase are essential for wounds (4). However, diabetic wounds often exhibit massive infiltration of inflammatory cells, sustained overexpression of pro-inflammatory cytokines (such as IL-1β, IL-18, and TNF-α), and abnormal activation of inflammation-related signaling pathways (5). This persistent inflammatory state prevents the wound from transitioning smoothly from the inflammatory phase to the proliferative phase, ultimately causing chronic non-healing wounds (6). Recent studies have shown that pyroptosis in macrophages plays a key role in regulating inflammation in diabetic wounds. The classical pyroptosis pathway relies on the assembly of the NLRP3 inflammasome and the activation of caspase-1 (7, 8). Activated caspase-1 cleaves gasdermin D (GSDMD), releasing its N-terminal domain with pore-forming activity, which creates pores in the cell membrane while promoting the maturation and release of related inflammatory factors (9). Therefore, targeting macrophage pyroptosis mediated by the NLRP3/Caspase-1/GSDMD pathway may be an effective strategy to promote the healing of diabetic wounds (10, 11).

Traditional Chinese medicine (TCM) has achieved remarkable progress in the clinical treatment of diabetic wounds (12). JDSJG, composed of four TCM herbs—scorpio, scolopendra, frankincense, and myrrh—possesses the therapeutic effects of clearing heat and detoxifying, promoting blood circulation and removing blood stasis, and removing necrotic tissue to promote tissue regeneration. Current clinical practice has confirmed that JDSJG can accelerate the healing of difficult-to-heal diabetic wounds (13). And animal studies have also shown that JDSJG intervention can reduce inflammation and promote wound healing (14). However, the specific molecular mechanism by which JDSJG promotes diabetic wound healing through the regulation of NLRP3/Caspase-1/GSDMD pathway-mediated macrophage pyroptosis remains unclear.

## 2 Materials and methods

### 2.1 Animals

Male C57BL/6 mice aged 8–10 weeks were purchased from Hunan Slaike Jingda Laboratory Animal Co., Ltd. (Laboratory Animal License No. SYXK (Xiang) 2020-0010). The study protocol was approved by the Animal Experiment Ethics Committee of Hunan University of Chinese Medicine (Ethics Approval No. 202409091). The animals were housed in an SPF-grade containment system at an ambient temperature of 25 °C under a 12-hour light/12-hour dark cycle. During the experiment, the animals had unrestricted access to food and water, with the exception of a single night of fasting prior to model induction. The entire experiment was conducted in strict accordance with the Guidelines for the Ethical Review of Laboratory Animal Welfare and relevant national regulations on laboratory animal management.

### 2.2 Preparation of Jiedu-Shengji Ointment

JDSJG was prepared by the First Affiliated Hospital of Hunan University of Traditional Chinese Medicine (Changsha, China). Ultrafine powder particles of scorpio, scolopendra, vinegar-processed frankincense, and myrrh were mixed with petroleum jelly in a 1:1:3:3 ratio to form an ointment, which was then sterilized before use.

JDSJG was formulated into a lyophilized powder for subsequent cellular experiments. The preparation process is as follows: scorpio, scolopendra, frankincense, and myrrh were weighed in a 1:1:3:3 ratio, water-extracted, and concentrated after alcohol precipitation. The concentrated herbal solution was pre-frozen overnight at -20 °C, and the crude extract of the compound was obtained after filtration. Next, the pre-frozen sample is rapidly transferred to a vacuum freeze-dryer (Ningbo SCIENTZ, SCIENTZ-30). It is freeze-dried under vacuum at -20 °C for 24 hours, followed by desorption drying at 30 °C for 12 hours, until the sample becomes a loose lump or powder. The dried material is ground into a fine powder, sieved through a 200-mesh screen, yielding the freeze-dried powder. Subsequently, a specific amount of the freeze-dried powder was weighed into a sterile centrifuge tube, mixed with sterile water o form a stock solution, filtered to remove bacteria, and stored at -80 °C for future use.

### 2.3 Preparation of a mouse diabetic wound model and drug treatment

All mice were raised for a one-week adaptive acclimatization period. Then, the diabetic mice were fed a high-sugar and high-fat diet for another 4 weeks. After 12 hours of fasting, streptozotocin (S8050, Solarbio) was administered intraperitoneally at a dose of 150 mg/kg. Random blood glucose levels measured via tail vein blood sampling over 3 consecutive days all exceeded 16.7 mmol/L, indicating successful establishment of the diabetic mouse model. After continuing the diet for 3 weeks, once blood glucose levels had stabilized, the mice were fasted for 12 hours and anesthetized via intraperitoneal injection of sodium pentobarbital (11715, Sigma-Aldrich) at a dose of 50 mg/kg. After shaving the lumbar and dorsal regions, a 0.7-cm-diameter punch was used in symmetrical positions on both sides of the bare dorsal area. In conjunction with ophthalmic scissors and curved forceps, the skin was incised along the marked lines down to the deep subcutaneous fascia to establish a diabetic wound model in mice (15).

Drug administration started on the day of modeling. The Control and Model groups received saline intervention. The JDSJG group underwent wound dressing with JDSJG, and the positive control HAMDY group underwent wound dressing with silver sulfadiazine ointment (National Drug Approval No. H44020614, Guangdong Hengjian Pharmaceutical Co., Ltd.). All dressings were applied once daily and secured with bandages. No signs of infection were observed in the wound sites of any group during this period, thereby ruling out the interference of infection on wound inflammatory markers.

### 2.4 Cell lines and cell culture

The mouse monocyte-macrophage leukemia cell line RAW264.7 was obtained from the Biological Cell Laboratory of the Advanced Research Center at Central South University (Changsha, China). After thawing, RAW264.7 cells were seeded in T25 culture flasks and supplemented with 3 mL of high-glucose DMEM medium (C11995500BT, Gibco) containing 10% fetal bovine serum (FBS) (A6901FBS-500, Invigentech), and placed in a CO_2_ incubator (Thermo, Steri-Cycle i160) at 37°C and 5% CO_2_ for culture. Once the cells reached approximately 80% confluence, they were passaged. Cells in the logarithmic growth phase were selected for subsequent experimental studies.

### 2.5 Cell viability assay

Cell viability was assessed using the CCK-8 assay. With the exception of the Control group, all other groups were treated with different drug interventions according to the experimental design and cultured for 24 hours to allow the drugs to take effect. After treatment, 10 μL of CCK-8 reagent (BMU106-CN, Abbkine) was added to each well according to the manufacturer’s protocol. The 96-well plate was then returned to the incubator for 30 minutes, and optical density was measured at 450 nm using a microplate reader (TECAN, Spark). Cell survival rates were calculated for each well.

### 2.6 Wound healing status and wound healing rate

To assess wound healing in mice, the wound healing status of mice in each group was observed and photographed on days 0, 3, 7, and 14 post-modeling. The area of the photographed wound was measured using the image analysis software ImageJ to calculate the wound healing rate. Wound healing rate = (Original wound area – Wound area at observation time point)/Original wound area × 100%.

### 2.7 Hematoxylin-Eosin staining

H&E staining was used to evaluate the microstructure and repair status of mouse wound tissue. After tissue collection, the samples were immersed in 4% paraformaldehyde and stored in the dark. The specimens were dehydrated using an ethanol concentration gradient, cleared with xylene, embedded in paraffin, and sectioned into 4-μm-thick sections. After dewaxing and rehydration of the tissue sections, they were stained using an H&E kit (G1120, Solarbio), mounted, and examined under a microscope.

### 2.8 Flow cytometry

Flow cytometry was used to detect the ratio of M1 to M2 macrophages. Tissue samples were collected from mouse wound sites, placed on ice, washed with phosphate-buffered saline (PBS), minced, and ground using a syringe. The tissues were resuspended in 1640 medium containing 10% FBS (PM150110, Procell). After filtration through a 70 μm cell strainer, the cell suspension was centrifuged to collect cell pellets. Alternatively, RAW264.7 cells in a stable growth phase were seeded in 6-well plates, and the cell pellets were collected 24 hours after group-specific interventions. Add PBS to the cell pellet to form a single-cell suspension, transfer to flow cytometry tubes, and perform multicolor antibody incubation and staining in sequence. For cells derived from animal tissues, use LIVE/DEAD Fixable Aqua Dead Cell Stain (L34966, Invitrogen), CD45 (561037, BD Biosciences), CD11b (557396, BD Biosciences), F4_80 (123113, BioLegend), CD86 (561963, BD Biosciences), and CD206 (568808, BD Biosciences) to identify macrophages in the wound and characterize their phenotypes. Since the RAW264.7 cell line consists of macrophages, a simplified protocol (LIVE/DEAD Fixable Aqua Dead Cell Stain, F4_80, CD86, CD206) was used to directly analyze changes in their phenotype. After staining, samples were analyzed using a flow cytometer (Beckman Coulter, CytoFLEX), and subsequent data analysis was performed using FlowJo software.

### 2.9 Enzyme-Linked Immunosorbent Assay

ELISA was used to detect the expression levels of the inflammatory cytokines IL-1β, IL-18, and TNF-α. Wound skin tissues were collected from mice, lysed and centrifuged to obtain tissue supernatants. Meanwhile, blood samples were collected from the retroorbital venous plexus, and serum samples were separated by centrifugation. RAW264.7 cells were seeded overnight, then subjected to group-specific interventions. After 24 hours, the cell culture supernatants from each group were collected by centrifugation. Following the instructions, the Mouse IL-1β One Step ELISA Kit (EK201BEG, MULTI SCIENCES), the Mouse IL-18 One Step ELISA Kit (EK218EGB, MULTI SCIENCES), and the Mouse TNF-α One Step ELISA Kit (EK282EGB, MULTI SCIENCES) to quantify the levels of IL-1β, IL-18, and TNF-α in the samples.

### 2.10 Western blotting

Collect skin tissue from the wound site of mice, then obtain protein samples through lysis and centrifugation. Determine protein concentration using the BCA Protein Assay Kit (EK-5001, ECOTOP SCIENTIFIC) and normalize the results. Mix the protein sample with 5× SDS loading buffer in the specified ratio, and boil in a water bath for 5–10 minutes to ensure complete protein denaturation. Prepare an SDS-PAGE gel, and load the denatured protein sample and Protein Marker (PM2510, Smobio) sequentially into the loading wells. Perform constant-voltage electrophoresis until the proteins are separated. Subsequently, transfer the gel and block it. Incubate at 4°C with NLRP3 Antibody (68102-1-Ig, Protientech), GSDMD-N Antibody (A22523, ABclonal), pro-Caspase-1 Antibody (AB179515, Abcam), Caspase-1 Antibody (81482-1-RR, Protientech), ASC Antibody (67494-1-Ig, Protientech), IL-1β Antibody (P50520-1R1F, Abmart), and iNOS Antibody (T55993F, Abmart) at 4°C overnight. After washing with TBST, incubate with HRP-conjugated secondary antibody (diluted 1:1000) at room temperature for 2 hours. Immerse the protein bands in ECL chemiluminescent solution and image using Image Lab software. β-actin (66009-1-Ig, Protientech) was used as an internal control.

### 2.11 RNA extraction and real-time quantitative polymerase chain reaction assay

Total RNA was extracted using a total RNA extraction kit (AG21023, Accurate Biology). RNA reverse transcription was performed using the Evo M-MLV Reverse Transcription Pre-mix Kit (AG11728, Accurate Biology). For RNA amplification, primers and ddH_2_O were mixed according to the protocol to form a primer mixture, and SYBR Green dye and cDNA were mixed according to the protocol to form a reaction mixture. The primer pairs are as follows: NLRP3: 5’-AAACCCACCAGTGTGCAAGA-3’ (left primer) and 5’-CAAAGGCCCCTTGTAGCTCA-3’ (right primer); GSDMD: 5’-TGCGTGTGACTCAGAAGACC-3’ (left primer) and 5’-CAAACAGGTCATCCCCACGA-3’ (right primer); Caspase-1: 5’-CGAGGGTTGGAGCTCAAGTT-3’ (left primer) and 5’-AGAAGTCTTGTGCTCTGGGC-3’ (right primer); TNF-α: 5’-CACGCTCTTCTGTCTACTGAACTTC-3’ (left primer) and 5’-CTTGGTGGTTTGTGAGTGTGAGG-3’ (right primer); IL-1β: 5’-CTCGCAGCAGCACATCAACAAG-3’ (left primer) and 5’-CCACGGGAAAGACACAGGTAGC-3’ (right primer); IL-18: 5’-ACTTTGGCCGACTTCACTGT-3’ (left primer) and 5’-CCTCGAACACAGGCTGTCTT-3’ (right primer). Amplification and fluorescence detection were performed using an RT-qPCR instrument (Roche, LightCycler 96). The intensity of the fluorescence signals was recorded, and the expression levels of the target genes were calculated using a relative quantification method.

### 2.12 Immunofluorescence

RAW264.7 cells were seeded in 24-well plates overnight. After 24 hours of group-specific treatment, the cells were fixed with 4% paraformaldehyde (ES-8100, ECOTOP SCIENTIFIC), permeabilized with 0.25% Triton X-100 (CT11451, Coolaber), and incubated overnight at 4°C with GSDMD-N Antibody and Caspase-1 Antibody. The samples were then stained with Cy3- or FITC-conjugated secondary antibodies. After incubation with DAPI, images were captured using a fluorescence microscope (Carl Zeiss, Axioscope 5), and fluorescence intensity was analyzed using ImageJ software.

### 2.13 LDH leakage assay

Collect the culture supernatants from cells subjected to different treatments. Using an LDH assay kit (A020-2, Nanjing Jiancheng Institute of Biological Engineering), mix the supernatant or lysate samples with the reaction buffer according to the instructions, and incubate at 37°C in the dark. Subsequently, measure the absorbance at 440 nm for each well using a microplate reader. Calculate the LDH activity of the cells in each well.

### 2.14 Scanning electron microscopy

Cell samples were fixed in 2.5% glutaraldehyde at 4°C for 4 hours, followed by sequential dehydration in a gradient of 30%, 50%, 70%, 80%, 90%, and 100% ethanol, with each step lasting 10 minutes. After dehydration, the samples were dried and coated with a layer of gold-palladium alloy approximately 10 nm thick. The treated samples were placed on the scanning electron microscope stage and observed and imaged under an acceleration voltage of 5–10 kV and at an appropriate working distance.

### 2.15 LC-MS/MS

Take an appropriate amount of sample, extract protein by ultrasonic centrifugation, precipitate protein with cold methanol, centrifuge and discard the supernatant; extract with acetonitrile solution containing HCl, lyophilize the supernatant after freezing and remove salts. Add an appropriate amount of 0.1% formic acid to dissolve the peptides, use a C18 desalting column (QL-C18-C-100) to desalt the sample, activate the column with 100%acetonitrile, equilibrate the column with 0.1% formic acid, load the sample onto the column, then wash the column with 0.1% formic acid to wash away impurities, finally elute with 40% acetonitrile, collect the flow-through liquid, and lyophilize. Nanoflow LC-MS/MS analysis of tryptic peptides was conducted on a quadrupole Orbitrap mass spectrometer (Orbitrap Exploris™480, Thermo Fisher Scientific, USA) coupled to an EASY nLC 1200 ultra-high pressure system (Thermo Fisher Scientific) via a nano-electrospray ion source.1ug of peptides were loaded on a 25 cm column (100 μm inner diameter, packed using ReproSil-Pur C18-AQ 1.5- µm silica beads; QL-HPLC-100*15). Peptides were separated using a gradient from 8 to 12% B in 5 min,then12% to 30% B in 33 min and stepped up to 40% in 7 min followed by a 15 min wash at 95% B at 300 nl per minute where solvent A was 0.1% formic acid in water and solvent B was 80% ACN and 0.1% formic acid in water. The total duration of the run was 60 min. Spectra were acquired with an Orbitrap Exploris™ 480 Mass Spectrometer (ThermoFisher Scientific) with FAIMS Pro™ Interface (ThermoFisher Scientific) cycling between CVs of -45 V and -65 V every 1 s. MS1 spectra were acquired at 120,000 resolution with a scan range from 350 to 1500 m/z, normalized AGC target of 3E6 and maximum injection time of 80 ms. MS2 normalized AGC target of 5E4 or maximum injection time (IT) of 22 ms fragmented with 27 normalized HCD collision energy and resulting spectra acquired at 15,000 resolution with a first mass of 200 m/z.

### 2.16 Database searches and *de novo* protein sequencing

RAW files were analyzed using the Peaks suite. The database selection was based on the target species, completeness of database annotations, and sequence reliability. The following principles were applied: for sequenced organisms, the species-specific database was used directly; for non-sequenced organisms, the most relevant broad-category proteome database was selected for the sample. The parameters were specified as follows: none enzyme, precursor ion mass tolerance of ±15 ppm, fragment ion mass tolerance of ±0.02 Da, and an auto local confidence (ALC) threshold of ≥50%. Percolator was used to filter peptide spectral matches and peptides to a false discovery rate (FDR) of less than 1%. After spectral assignment, each validated peptide sequence was aligned against the target protein database, and all protein entries that the peptide matched were recorded. Peptides were classified based on their uniqueness: peptides matching only one protein were designated as unique peptides, while those matching multiple proteins were designated as shared peptides. In accordance with the parsimony principle, proteins with identical peptide sets were classified into the same protein group. A protein inference algorithm was further used to define shared peptides as razor peptides and assign them to the protein group with the largest number of unique peptides. Within each protein group, a “master protein” was selected as the representative according to the default rule: the protein with the highest number of unique peptides, and in case of a tie, the protein with the smallest percent peptide coverage (i.e., the longest protein). Subsequently, the confidence at the protein level was calculated based on the combined probability of all constituent peptides within each protein group, and proteins were further filtered to achieve a final protein-level FDR of 1%. For quantitative analysis, only unique peptides and razor peptides were considered. These were aggregated to calculate the relative abundance of each protein group.

### 2.17 GO enrichment analysis and KEGG pathway enrichment analysis

The identified protein IDs were converted to Gene Symbols using the UniProt database. These data were then imported into the DAVID database (https://davidbioinformatics.nih.gov/summary.jsp) for GO and KEGG enrichment analysis. Entries showing significant enrichment were selected using a significance threshold of *P* < 0.05. Finally, the results of the enrichment analysis were visualized using the online bioinformatics platform (https://www.bioinformatics.com.cn/).

### 2.18 Statistical analysis

Data are presented as mean ± standard deviation ( $\bar{x}\pm \text{s}$). Results were analyzed statistically and plotted using GraphPad Prism 9.0. Each experiment was repeated at least three times. Significant differences between groups were analyzed using one-way or two-way analysis of variance (ANOVA) or paired t-tests; *P* < 0.05 was considered statistically significant.

## 3 Results

### 3.1 JDSJG promotes wound healing in STZ-induced diabetic mice

**Figure 1:**
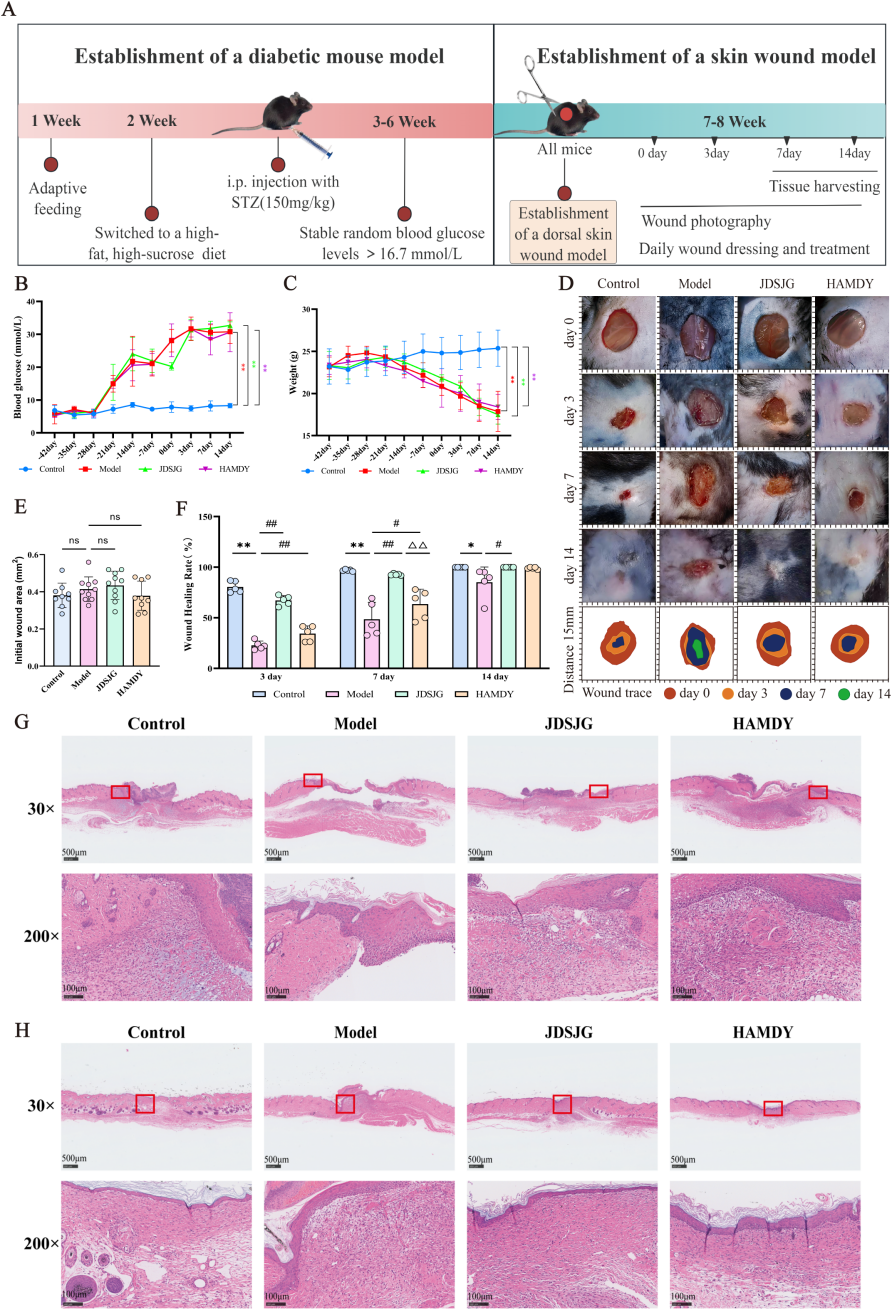
Effects of JDSJG on wound healing in STZ-induced diabetic mice. **(A)** Flowchart of the establishment and intervention of the diabetic wound model in mice. **(B, C)** Changes in blood glucose and body weight in mice over time. Statistical analysis was performed using two-way ANOVA (n = 5); data are expressed as mean ± standard deviation (x ± s). Day 0 corresponds to skin wound modeling; day –7 corresponds to 7 days prior to skin wound modeling; day 7 corresponds to 7 days after skin wound modeling; and so on. **(D)** Representative wound images at 0, 3, 7, and 14 days post-wound induction. **(E)** Initial wound area at the time of diabetic wound induction (i.e., on day 0). Statistical analysis was performed using one-way ANOVA (n = 9); data are presented as mean ± standard deviation (x ± s). **(F)** Changes in wound healing rates in mice over time. Statistical analysis was performed using two-way ANOVA (n = 5), and data are expressed as mean ± standard deviation (x ± s). **(G, H)** Representative H&E-stained skin sections from mice in each group at 7 and 14 days. ns indicates no significant difference (p > 0.05); *P < 0.05, **P < 0.01 vs. Control; #*P* < 0.05, ##*P* < 0.01 vs. Model; △△*P* < 0.01 vs. JDSJG.

To evaluate the efficacy of JDSJG on diabetic wounds, we established a mouse model of diabetes by combining STZ with a high-sugar, high-fat diet. Then, we created a skin wound model in these mice and applied group-specific interventions, as illustrated in **Figure 1A**. **Figures 1B, C** show changes in blood glucose and body weight over time. The results indicate that after STZ induction, blood glucose levels in the mice continued to rise and remained stable at a high level of >16.7 mmol/L. Also, as blood glucose levels fluctuated, the mice’s body weight showed a downward trend. These findings suggest successful establishment of the diabetic model. **Figure 1D** illustrates the wound healing process in each group of mice. There were no intergroup differences in initial wound area (**Figure 1E**). Compared with the normal group, the model group exhibited significantly delayed wound healing (**Figure 1F**). This is consistent with the known characteristics of impaired wound healing in diabetes (16–18). Notably, the application of JDSJG accelerated wound healing in diabetic mice (**Figures 1D–F**). These data indicate that JDSJG effectively promotes wound repair in diabetes. H&E staining revealed that 7 days after treatment, the Model group exhibited poor wound healing. This was characterized by loose tissue structure, discontinuous scab formation, slow epithelial migration, and extensive inflammatory cell infiltration. The healing process was significantly accelerated in the Control and JDSJG groups (showing wound contraction, active epithelialization, abundant collagen deposition, and mild inflammation), while the HAMDY group fell between the two (**Figure 1G**).After 14 days of treatment, the wounds in the Control and JDSJG groups were completely healed. They had matured and thinned epidermis, clear dermal structure, and visible new hair follicles and sebaceous glands. Although the wound in the HAMDY group had closed, the epithelial layer remained thick, suggesting it was in the remodeling phase. The Model group exhibited delayed healing with incomplete epithelial coverage, residual scabs in some areas, and accompanying inflammation and erythrocyte exudation (**Figure 1H**). In summary, JDSJG significantly promotes wound healing in diabetic mice.

### 3.2 JDSJG improves ultrastructural damage in wounds of diabetic mice

**Figure 2:**
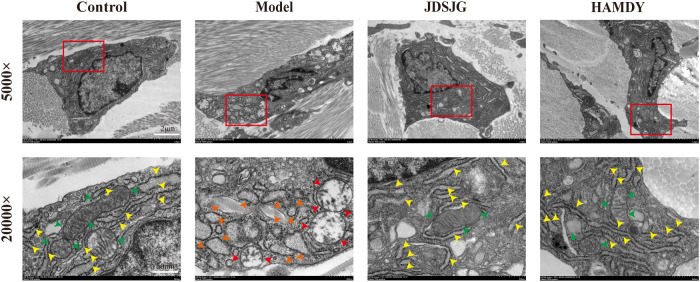
Effects of JDSJG on the ultrastructure of wounds in diabetic mice. Magnifications are 5000× and 20000×. The green arrows indicate normal mitochondria;the yellow arrows indicate normal endoplasmic reticulum the red arrows indicate swollen mitochondria;the orange arrows indicate swollen endoplasmic reticulum.

Damage to organelles can lead to impaired energy metabolism and disrupted protein synthesis, constituting a key pathological basis for delayed wound healing (19). Transmission electron microscopy (TEM) results are shown in **Figure 2**. Ultrastructural damage was observed in mitochondria and the endoplasmic reticulum (ER) of diabetic wounds, primarily manifested as mitochondrial cristae fragmentation and vacuolization, ER dilation, and ribosomal detachment. Following intervention with JDSJG and HAMDY, the aforementioned ultrastructural damage was significantly improved.

### 3.3 JDSJG promotes M2 macrophage polarization

**Figure 3:**
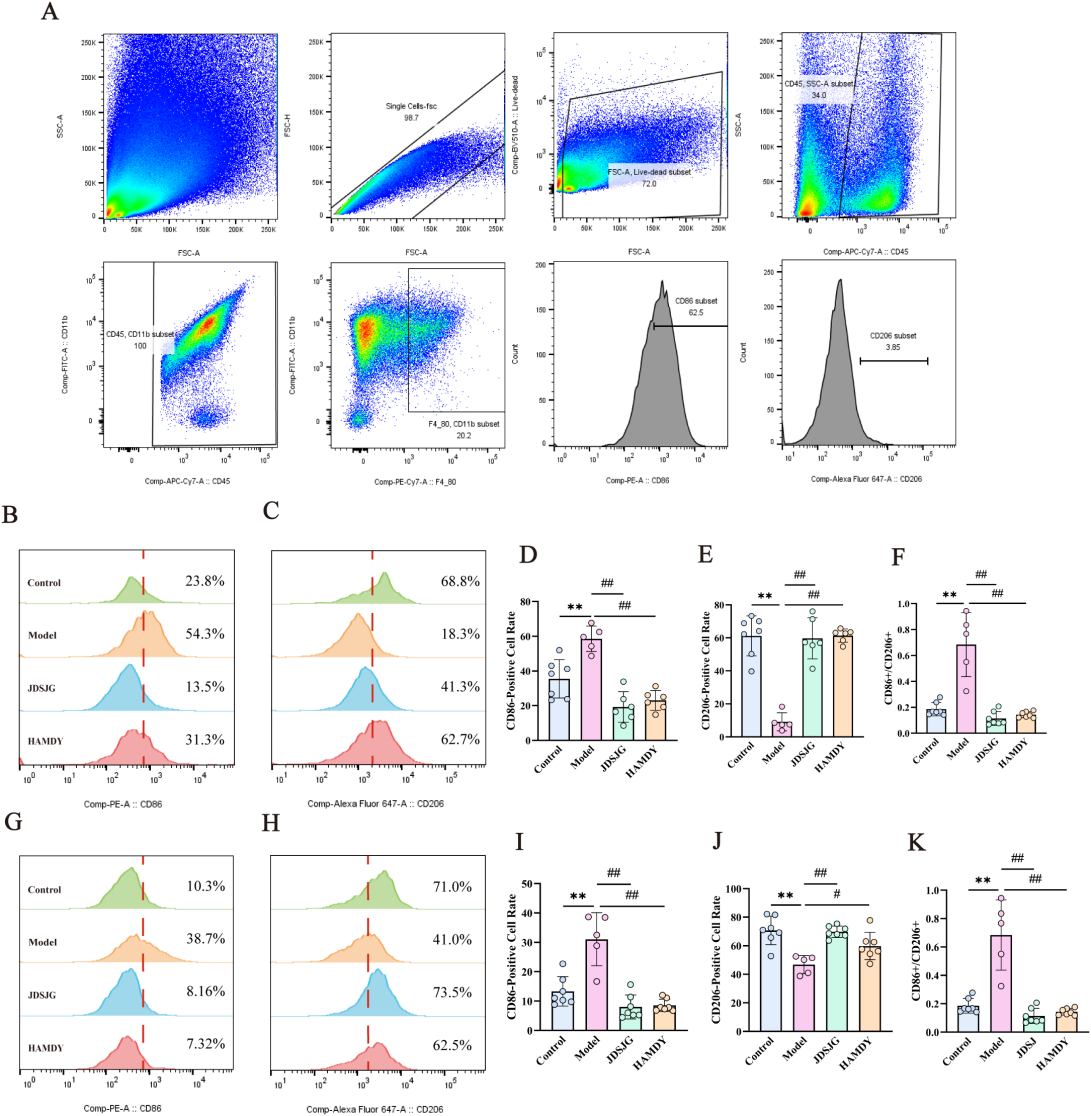
Effects of JDSJG on the phenotype of macrophages in wound tissue of diabetic mice. **(A)** Flow cytometric identification of macrophages using a gating strategy. **(B, C)** Bar charts showing the proportions of M1 and M2 macrophages in wound tissue from mice in each group on day 7. **(D, E)** Quantitative analysis of the proportions of M1 and M2 macrophages in wound tissue from mice in each group on day 7 (n = 5, x ± s). **(F)** Changes in the M1/M2 macrophage ratio in wound tissue from mice on day 7 (n = 5, x ± s). **(G, H)** Bar charts showing the proportions of M1 and M2 macrophages in wound tissues of mice in each group on day 14. **(I, J)** Quantitative analysis of the proportions of M1 and M2 macrophages in wound tissues of mice in each group on day 14 (x ± s). **(K)** Changes in the M1/M2 macrophage ratio in mouse wound tissue on day 14 (x ± s). Statistical analysis was performed using one-way ANOVA. **P < 0.01 vs. Control; #*P* < 0.05, ##*P* < 0.01 vs. Model.

Next, we used flow cytometry to detect changes in the phenotype of wound macrophages, with the gating strategy shown in **Figure 3A**. The results indicated that on day 7 after wound modeling, compared with the Control group, the Model group exhibited a significantly higher proportion of M1-type macrophages, a significantly lower proportion of M2-type macrophages, and a sharp increase in the M1/M2 ratio (**Figures 3B–F**). Treatment with JDSJG and HANDY significantly reduced the proportion of M1 macrophages, increased the proportion of M2 macrophages, and promoted the M1/M2 ratio to approach normal levels (**Figures 3B–F**). The results on day 14 were consistent with those on day 7 (**Figures 3G–K**). These findings suggest that JDSJG may improve the M1/M2 imbalance by regulating the polarization of macrophages in wound tissue from diabetic mice toward the M2 phenotype.

### 3.4 JDSJ inhibits the expression of inflammatory factors in diabetic mice

**Figure 4:**
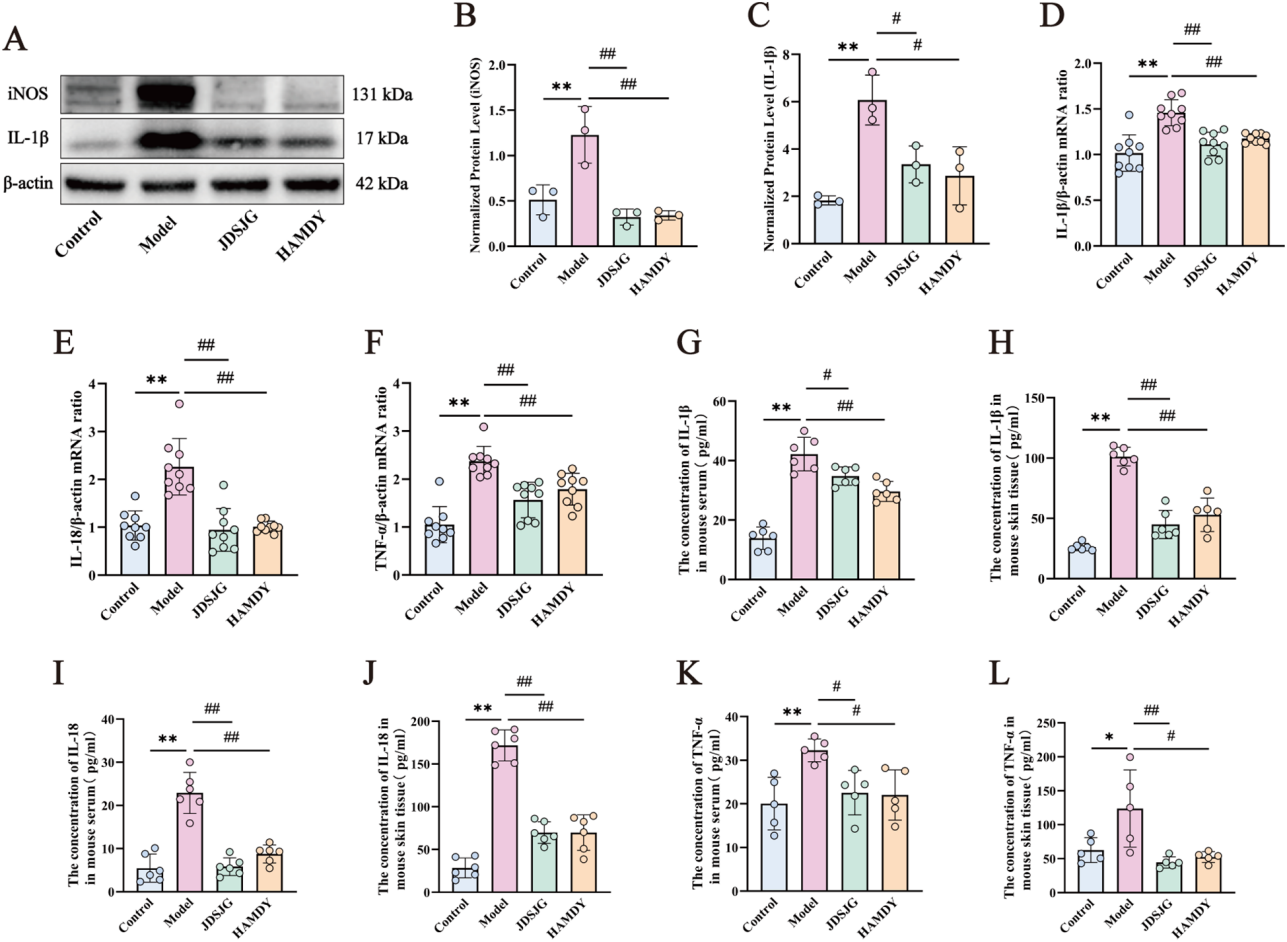
Effects of JDSJG on the expression of inflammatory factors in diabetic mice. **(A)** Representative images of iNOS and IL-1β proteins (n=3). **(B, C)** Quantitative analysis of iNOS and IL-1β protein expression ( $\bar{x}\pm \text{s}$, n=3). **(D–F)** RT-qPCR analysis of IL-1β, IL-18, and TNF-α mRNA expression levels in wound tissue ( $\bar{x}\pm \text{s}$, n=9). **(G–I)** ELISA analysis of inflammatory factor levels (IL-1β, IL-18, and TNF-α) in mouse serum ( $\bar{x}\pm \text{s}$, n=6). **(J–L)** Expression levels of inflammatory factors IL-1β, IL-18, and TNF-α in mouse skin tissue, as determined by ELISA ( $\bar{x}\pm \text{s}$, n=6).Statistical analysis was performed using one-way ANOVA. *P < 0.05, **P < 0.01 vs. Control; #*P* < 0.05, ##*P* < 0.01 vs. Model.

To evaluate the effect of JDSJG on the inflammatory response in a diabetic mouse wound model, we used Western blot, real-time quantitative PCR, and ELISA to detect the expression levels of inflammation-related factors. Western blot results showed that, compared with the Control group, the protein expression levels of iNOS and IL-1β in wound tissues of the Model group mice were significantly elevated. However, intervention with JDSJG and HAMDY effectively reversed this trend (**Figures 4A–C**). Next, we detected the expression levels of the IL-1β, IL-18, and TNF-α genes via RT-qPCR. The results indicated that the mRNA expression levels of IL-1β, IL-18, and TNF-α were all significantly upregulated in the wound tissue of the Model group. Furthermore, the transcriptional levels of these inflammatory factors decreased after drug intervention (**Figures 4D–F**). To further investigate the anti-inflammatory effects of JDSJG, we used ELISA to detect changes in the concentrations of the inflammatory factors IL-1β, IL-18, and TNF-α in mouse serum and skin tissue. These results were consistent with those from RT-qPCR (**Figures 4G–L**). In summary, JDSJG exhibits significant anti-inflammatory effects in diabetic wounds.

### 3.5 JDSJG inhibits the expression of proteins associated with the NLRP3/Caspase-1/GSDMD signaling pathway

**Figure 5:**
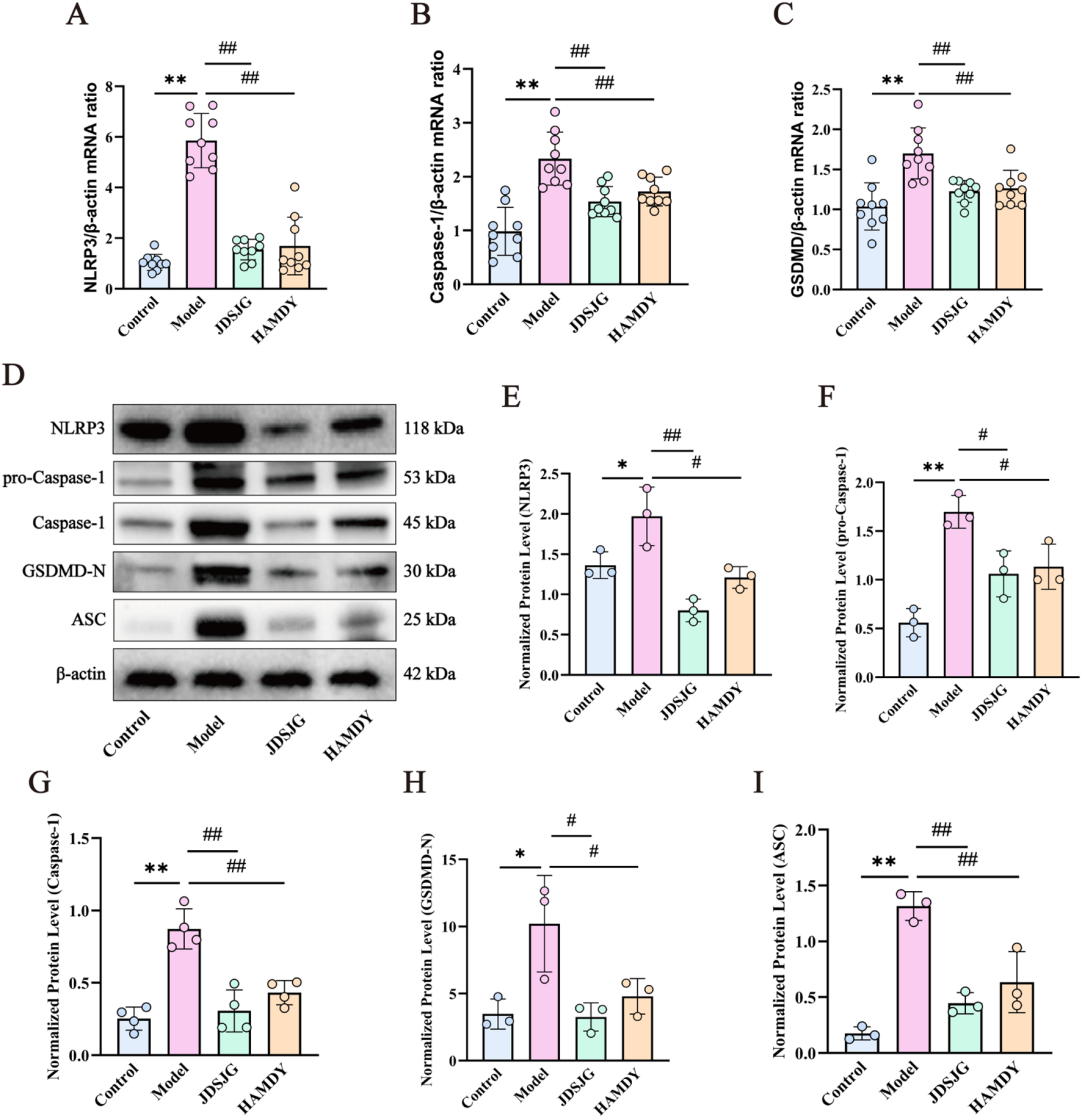
Regulatory effects of JDSJG on the NLRP3/Caspase-1/GSDMD signaling pathway. **(A–C)** RT-qPCR analysis of NLRP3, Caspase-1, and GSDMD mRNA expression levels in wound tissue ( $\bar{x}\pm \text{s}$, n = 9). **(D)** Representative images of NLRP3, pro-Caspase-1, Caspase-1, GSDMD-N, and ASC proteins (n = 3). **(E–I)** Quantitative analysis of NLRP3, pro-Caspase-1, Caspase-1, GSDMD-N, and ASC protein expression ( $\bar{x}\pm \text{s}$, n = 3).Statistical analysis was performed using one-way ANOVA. *P < 0.05, **P < 0.01 vs. Control; #*P* < 0.05, ##*P* < 0.01 vs. Model.

The NLRP3/Caspase-1/GSDMD signaling pathway is a classic pyroptosis pathway (20–22). This study further investigated the effects of JDSJG on the NLRP3/Caspase-1/GSDMD signaling pathway. Compared with the Control group, the Model group showed significantly increased levels of NLRP3, Caspase-1, and GSDMD mRNA (*P* < 0.01), which were restored to normal levels after JDSJG treatment (*P* < 0.05, **Figures 5A–C**). Concurrently, Western blot results showed that, compared with the Control group, the Model group exhibited significantly elevated levels of NLRP3, pro-Caspase-1, Caspase-1, GSDMD-N, and ASC proteins. This indicates that this signaling pathway is abnormally activated under diabetic conditions. However, following JDSJG intervention, the gene and protein expression of these molecules was suppressed (see **Figures 5D–I**). These results suggest that JDSJG can inhibit the activation of the NLRP3/Caspase-1/GSDMD signaling pathway.

### 3.6 JDSJG exerts a protective effect on LPS-induced RAW264.7 macrophages

**Figure 6:**
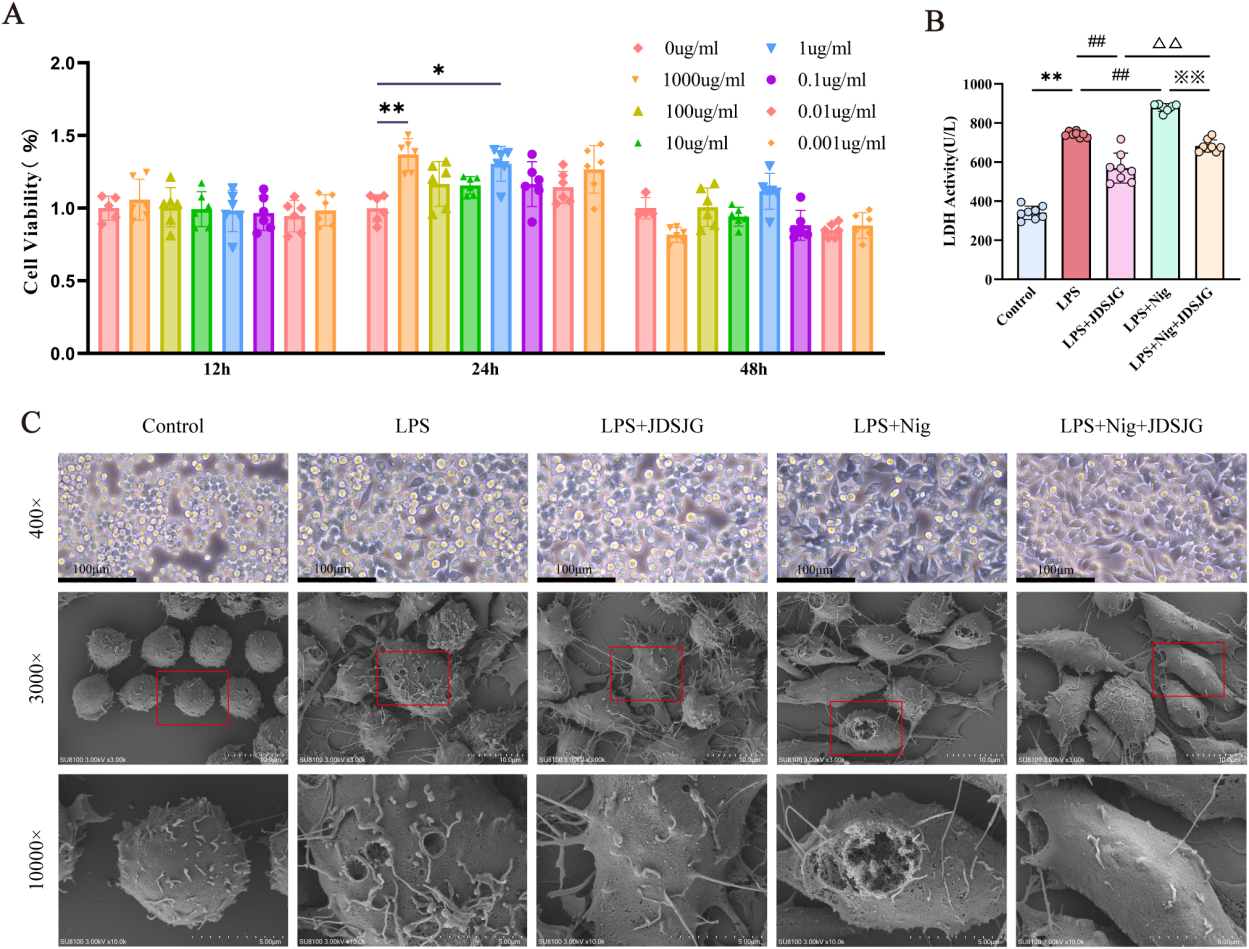
Effects of JDSJG on LPS-induced RAW264.7 macrophages. **(A)** The effects of different concentrations of JDSJG on cell viability at various time points were assessed using the CCK-8 assay. Statistical analysis was performed using two-way analysis of variance (n = 6), and data are presented as mean ± standard deviation (x ± s). **(B)** Changes in LDH release following group-specific interventions were assessed using a microplate assay. Statistical analysis was performed using one-way ANOVA (n = 8), and data are presented as mean ± standard deviation (x ± s). **(C)** Changes in cell morphology were observed using electron microscopy and scanning electron microscopy. *P < 0.05, **P < 0.01 vs. Control; ##*P* < 0.01 vs. LPS; △△*P* < 0.01 vs. LPS+JDSJG; ※※*P* < 0.01 vs. LPS+Nig.

Next, we further investigated the effects of JDSJG through *in vitro* experiments. First, we used the CCK-8 assay to screen for the optimal concentration and duration of JDSJG treatment. As shown in **Figure 6A**, we ultimately selected a concentration of 1 μg/ml and a treatment duration of 24 hours for subsequent experiments. After treatment, cell culture supernatants and cell pellets were collected from each group for analysis. As shown in **Figure 6B**, LPS stimulation caused a significant increase in LDH release. LDH release further increased following treatment with nigericin. However, intervention with JDSJG resulted in a marked reduction in LDH release levels. Electron microscopy and scanning electron microscopy observations (**Figure 6C**) revealed that under the influence of LPS and nigericin, cells exhibited typical pyroptotic morphological changes. These changes included cell swelling, deformation, and the formation of membrane pores, indicating compromised cell membrane integrity. In contrast, following intervention with the JDSJG, the morphological damage to the cells was significantly improved. These results indicate that JDSJG exerts a protective effect on cells and can mitigate cellular damage.

### 3.7 JDSJG promotes the polarization of RAW264.7 cells toward the M2 phenotype

**Figure 7:**
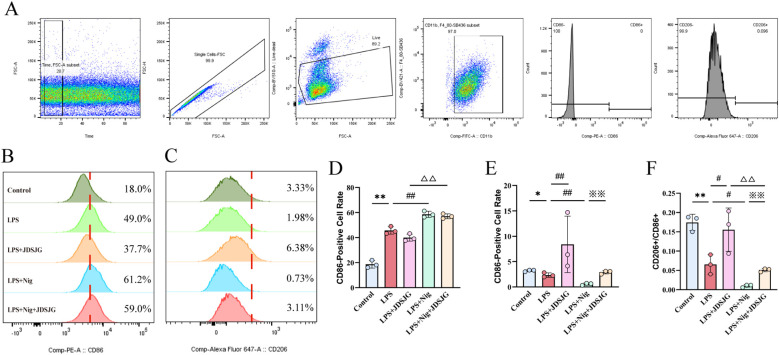
Effects of JDSJG on LPS-induced RAW264.7 macrophages. **(A)** Flow cytometric analysis of macrophage polarization using gating strategies. **(B, C)** Box-and-whisker plots showing the proportions of M1 and M2 macrophages. **(D, E)** Quantitative analysis of changes in the proportions of M1 and M2 macrophages ( $\bar{x}\pm \text{s}$, n = 3). **(F)** Changes in the M2/M1 macrophage ratio (n = 3).Statistical analysis was performed using one-way ANOVA. *P < 0.05, **P < 0.01 vs. Control; #*P* < 0.05, ##*P* < 0.01 vs. LPS; △△*P* < 0.01 vs. LPS+JDSJG; ※※*P* < 0.01 vs. LPS+Nig.

To investigate the regulatory role of JDSJG in macrophage polarization, we used flow cytometry to detect phenotypic changes in RAW264.7 cells under different treatment conditions. First, we established a reasonable gating strategy based on cell morphology and fluorescent markers. This strategy excluded cell debris and adherent particles to obtain the target cell population for analysis, as shown in **Figure 7A**. Flow cytometry results (**Figures 7B–E**) showed that under the influence of LPS and nigericin, the proportion of M1-type macrophages (CD86^+^) significantly increased, while the proportion of M2-type macrophages (CD206^+^) correspondingly decreased. After JDSJG intervention, there was no significant statistical difference in the proportion of M1 macrophages, but the proportion of M2 macrophages showed a significant rebound. We further analyzed changes in the M2/M1 ratio to assess the overall trend of macrophage polarization. As shown in **Figure 7F**, following LPS and nigericin intervention, the M2/M1 ratio decreased significantly. This suggests an intensified polarization of macrophages toward the M1 phenotype. However, after JDSJG intervention, the M2/M1 ratio rebounded markedly. These results indicate that JDSJG promotes the conversion of macrophages to the M2 phenotype and regulates the M1/M2 balance. This may be one of the key mechanisms by which it promotes diabetic wound healing.

### 3.8 Inhibition of inflammatory cytokine expression in RAW264.7 cells by JDSJG

**Figure 8:**
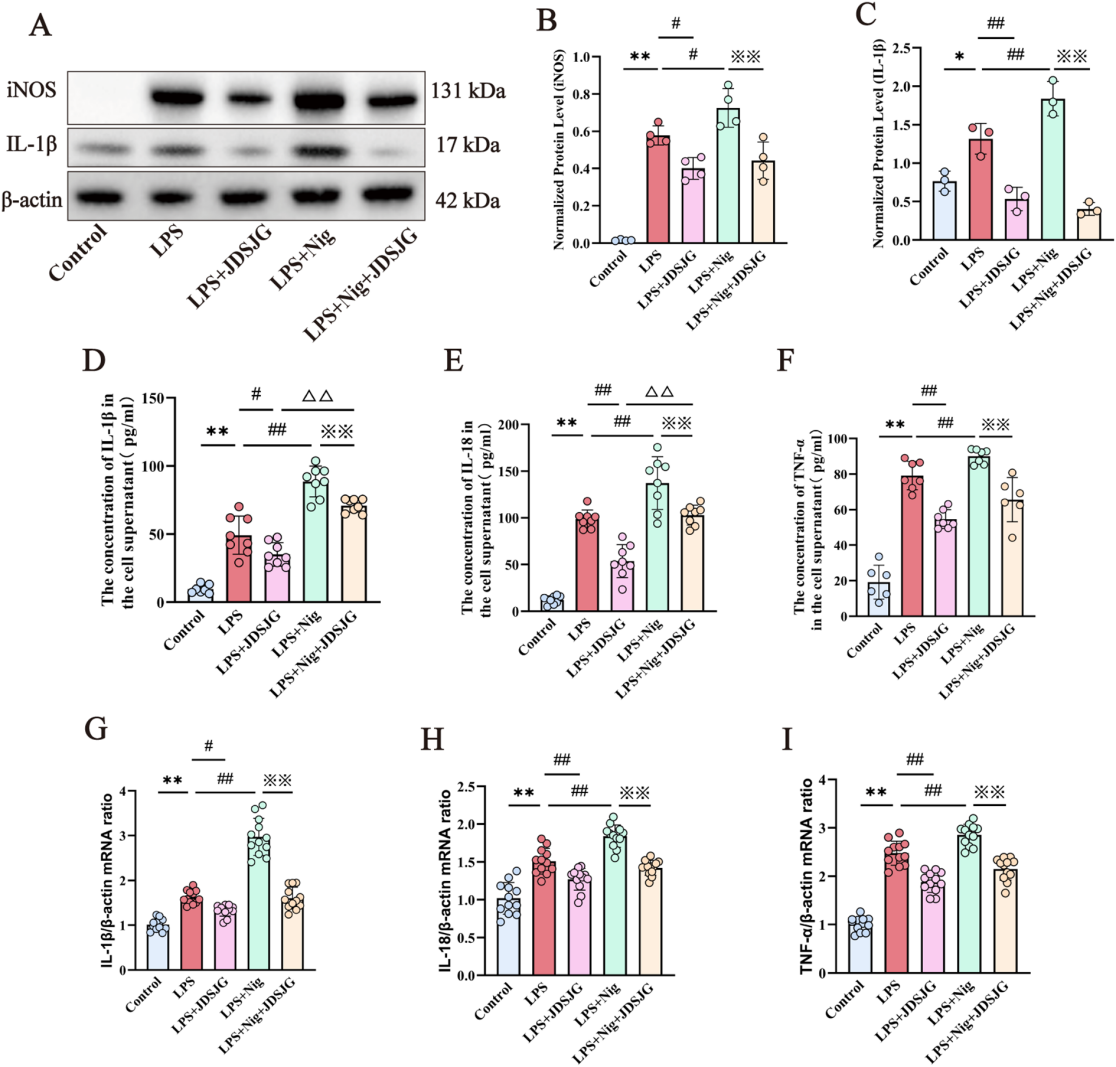
Effects of JDSJG on the expression of inflammatory cytokines in RAW264.7 cells. **(A)** Representative images of iNOS and IL-1β proteins (n = 3). **(B, C)** Quantitative analysis of iNOS and IL-1β protein expression ( $\bar{x}\pm \text{s}$, n = 3). **(D–F)** Expression levels of the inflammatory cytokines IL-1β, IL-18, and TNF-α in cell culture supernatants, as determined by ELISA ( $\bar{x}\pm \text{s}$). **(G–I)** mRNA expression levels of IL-1β, IL-18, and TNF-α in wound tissue, as determined by RT-qPCR ( $\bar{x}\pm \text{s}$, n = 9). Statistical analysis was performed using one-way ANOVA. *P < 0.05, **P < 0.01 vs. Control; #*P* < 0.05, ##*P* < 0.01 vs. LPS; △△*P* < 0.01 vs. LPS+JDSJG; ※※*P* < 0.01 vs. LPS+Nig.

To investigate the effects of JDSJG on the inflammatory response in RAW264.7 cells, we used ELISA, Western blot, and RT-qPCR to detect the protein and gene expression levels of inflammatory factors. The results of Western blot and quantitative analysis (**Figures 8A–C**) showed that JDSJG significantly inhibited the LPS- and Nigericin-induced upregulation of iNOS and IL-1β protein expression. ELISA results showed (**Figures 8D–F**) that following LPS and Nigericin treatment, protein levels of IL-1β, IL-18, and TNF-α increased significantly. Furthermore, JDSJG partially inhibited this upward trend. RT-qPCR results corroborated the ELISA findings (**Figures 8G–I**). In summary, these findings confirm that JDSJG inhibits the expression of inflammatory cytokines in macrophages induced by LPS and Nigericin, thereby exerting an anti-inflammatory effect.

### 3.9 JDSJG inhibits the expression of the NLRP3/Caspase-1/GSDMD signaling pathway in RAW264.7 cells

**Figure 9:**
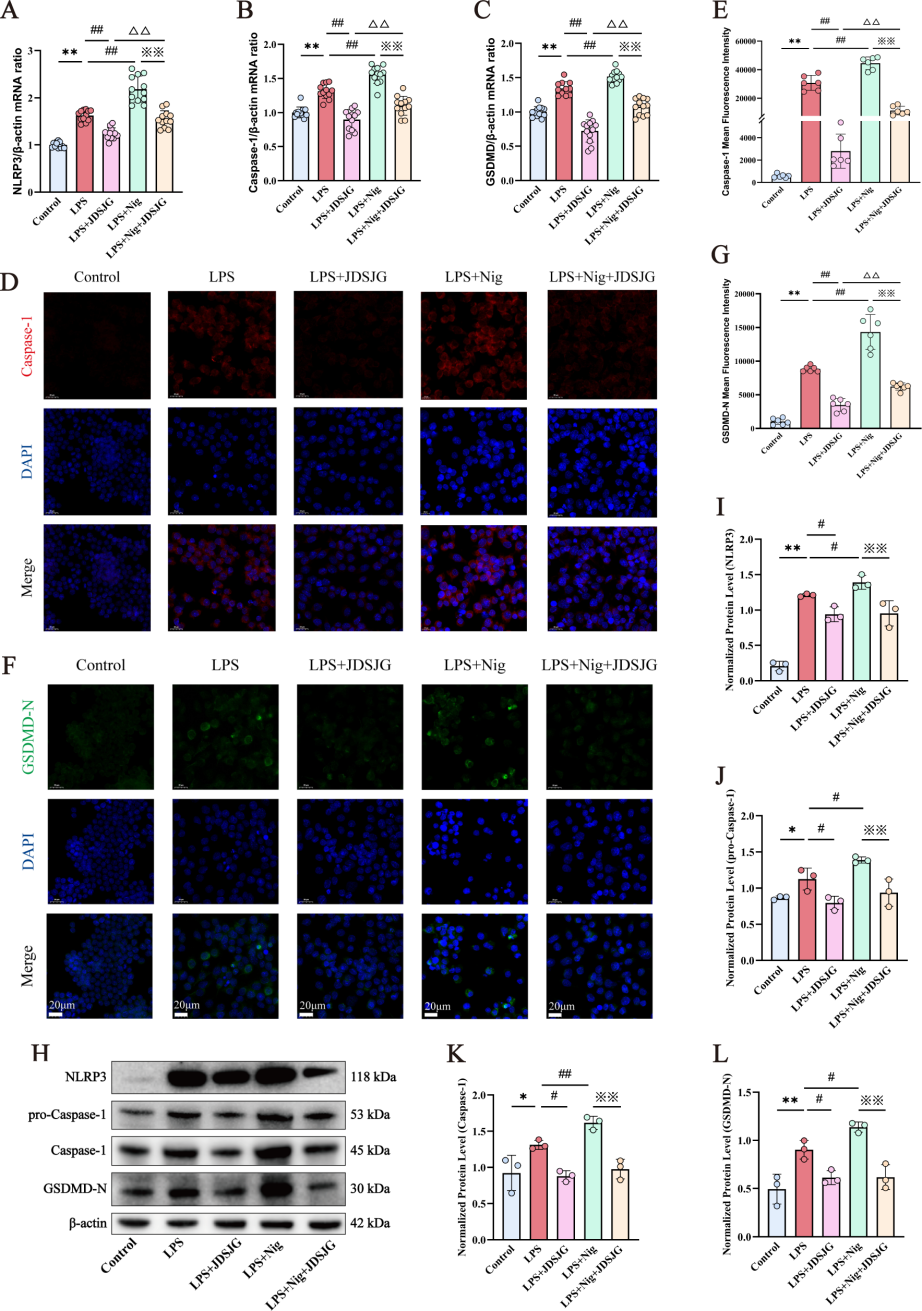
Regulatory effects of JDSJG on the NLRP3/Caspase-1/GSDMD signaling pathway in RAW264.7 cells. **(A–C)** Expression levels of NLRP3, Caspase-1, and GSDMD in RAW264.7 cells as determined by RT-qPCR ( $\bar{x}\pm \text{s}$, n = 12). **(D, E)** Representative images of Caspase-1 immunofluorescence and bar charts of quantitative analysis. **(F, G)** Representative images of GSDMD-N immunofluorescence and bar charts of quantitative analysis. **(H)** Representative protein bands of NLRP3, pro-Caspase-1, Caspase-1, and GSDMD-N. **(I–L)** Bar charts showing quantitative analysis of NLRP3, pro-Caspase-1, Caspase-1, and GSDMD-N protein levels ( $\bar{x}\pm \text{s}$, n = 3). Statistical analysis was performed using one-way ANOVA. *P < 0.05, **P < 0.01 vs. Control; #*P* < 0.05, ##*P* < 0.01 vs. LPS; △△*P* < 0.01 vs. LPS+JDSJG; ※※*P* < 0.01 vs. LPS+Nig.

To further validate the regulatory role of JDSJG on the pyroptosis pathway, we performed RT-qPCR, immunofluorescence, and Western blot assays. These assays detected the expression of key molecules in the NLRP3/Caspase-1/GSDMD pathway. RT-qPCR results (**Figures 9A–C**) showed that the gene expression levels of NLRP3, Caspase-1, and GSDMD were significantly elevated following stimulation with LPS and nigericin. In contrast, JDSJG intervention effectively downregulated their expression levels. Immunofluorescence staining and quantitative analysis indicated (**Figures 9D–G**) that both LPS and Nigericin stimulation led to increased fluorescence intensity of Caspase-1 and GSDMD-N. However, JDSJG intervention significantly reversed this change. Western blot and quantitative analysis further confirmed (**Figures 9H–L**) that JDSJG can inhibit the LPS- and Nigericin-induced upregulation of NLRP3, pro-Caspase-1, Caspase-1, and GSDMD-N protein expression. In summary, JDSJG may exert its anti-inflammatory and pyroptosis-regulating effects by inhibiting the abnormal activation of the NLRP3/Caspase-1/GSDMD signaling pathway induced by LPS and nigericin.

### 3.10 Analysis of biopeptide information in scorpio and scolopendra

**Figure 10:**
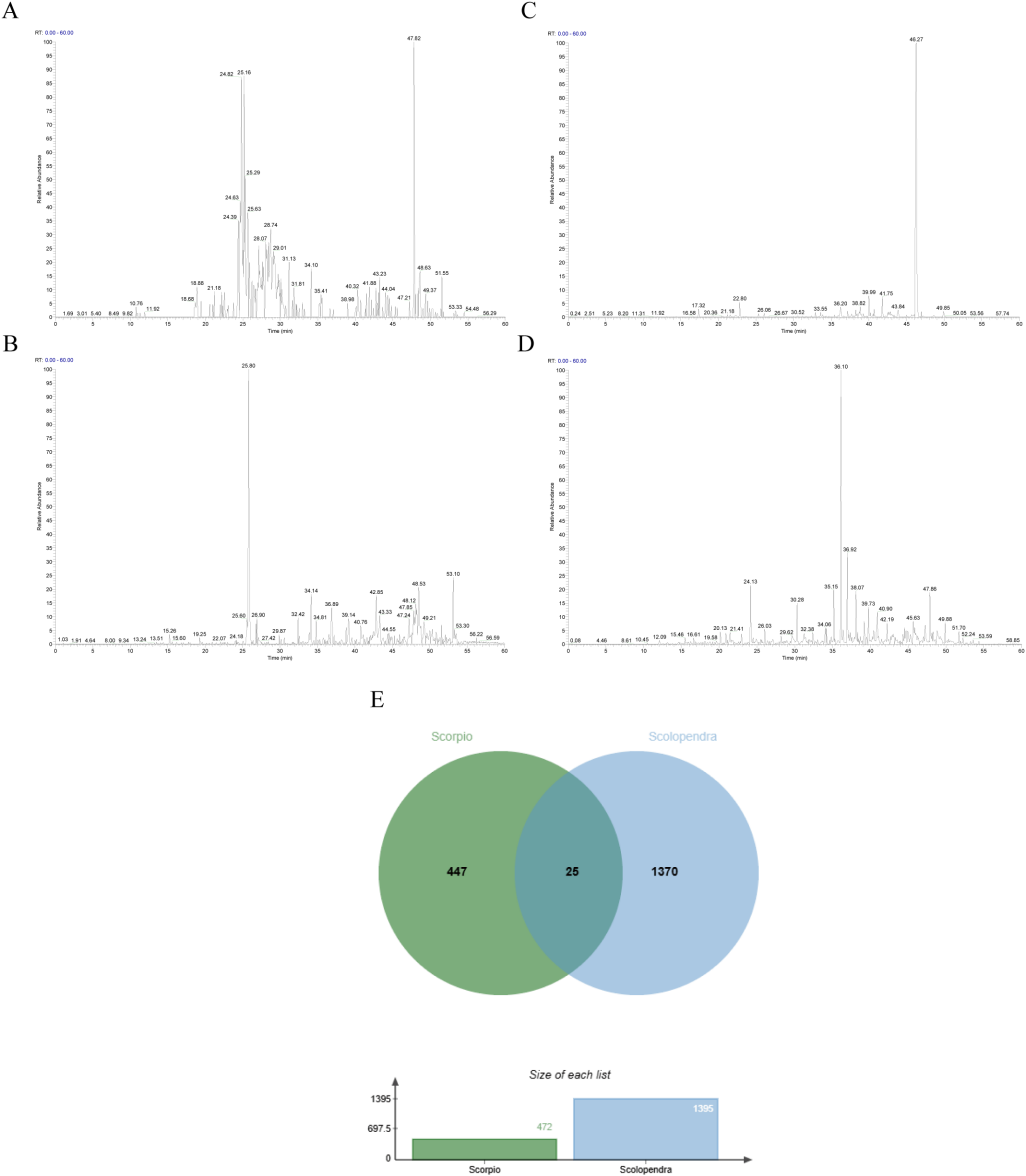
FAIMS base peak chromatograms of scorpio and scolopendra. **(A, B)** Base peak chromatograms (BPC) of scorpio samples under FAIMS compensation voltages of –65 V **(A)** and –45 V **(B)**. **(C, D)** Base peak chromatograms (BPC) of scolopendra samples under FAIMS compensation voltages of –65 V **(C)** and –45 V **(D)**. **(E)** Venn diagram showing the number of peptide segments in scorpio and scolopendra.

Previous experiments have confirmed that JDSJG promotes the healing of diabetic wounds, but its underlying mechanism remains unclear. We used LC-MS/MS combined with FAIMS (alternating compensation voltages of -65 V and -45 V) to collect mass spectrometry data from scorpio and scolopendra. This generated base peak chromatograms (**Figures 10A–D**). Using Peaks 8 software for *de novo* peptide sequencing and sequence homology searches, we identified 472 peptides in the scorpio sample and 1,395 peptides in the scolopendra samples. Among these, 25 peptides shared between the two (**Figure 10E**).The peptide results were entered into PeptideRanker (http://distilldeep.ucd.ie/peptideranker) to predict the potential biological activity of the peptides. The results showed that among the scorpio peptides, a total of 100 peptides had a PR value ≥ 0.50, of which 33 had a PR value ≥ 0.80. Among the scolopendra peptide segments, a total of 142 had PR values ≥ 0.50, with 10 of them having PR values ≥ 0.80. Subsequently, the basic characteristics of the peptides with PR values ≥ 0.80 were summarized. The results showed that the molecular weights of these peptides ranged from 451 to 2747 Da, and their sequence lengths ranged from 5 to 29 amino acids, as detailed in **Table 1**.

**Table 1:** Basic characteristics of peptides with PR value ≥ 0.80.

Peptide	Source	-10LgP	Mass	Length	ppm	m/z	z	RT	Area Scol
PPPPPPPP	SC	25.94	794.4326	8	5.6	398.22583	2	22.2026	181000
GYLGLGYGGLYGAYGGLYGAGVPVGR	SC	36.58	2506.2644	26	6.2	836.43396	3	51.7439	242000
FRFGSF	SC	33.49	759.3704	6	6.9	380.6951	2	42.2462	155000
GYLGLGYGGLYGAYGGLYGAGVPVGRAVA	SC	36.58	2747.407	29	6.8	916.81586	3	52.4885	177000
GHLFKLATKIIPSLFRRKNQRS	SC	36.58	2609.5393	22	-0.7	522.91479	5	45.1897	400000
FFNILR	SC	24.74	808.4595	6	1.3	405.23758	2	44.3088	124000
GFGGGRGGFGGGRG	SC	26.29	1194.5642	14	2.4	399.19629	3	19.5542	122000
GGFGGGRGGFGGG	SC	36.58	1038.4631	13	0.6	520.23914	2	23.8184	176000
GFGGGRGGFGGGR	SC	36.46	1137.5427	13	0	380.18817	3	19.217	59100
GGFGGGRGGF	SC	25.3	867.3987	10	1.2	434.70715	2	26.3601	92500
FFGHLFKLA	SC	35.49	1078.5963	9	0.6	540.30579	2	46.5316	268000
GGFGGGRGGFGG	SC	36.56	981.4417	12	-0.2	491.728	2	24.3173	382000
GGFGGGRGGFG	SC	36.58	924.4202	11	1.7	463.21814	2	25.1576	337000
GFGGGRGGFGGG	SC	36.08	981.4417	12	1.2	491.72867	2	23.383	96700
GYYGLRYF	SC	36.29	1037.4971	8	4.1	1038.50854	1	46.1294	34200
GFGGGRGGFGG	SC	34.12	924.4202	11	0.9	463.21777	2	24.0213	378000
GFGGGRGGF	SC	26.43	810.3773	9	3.4	406.19727	2	26.0898	70300
YGGFGKFH	SC	36.43	911.429	8	1.4	456.72241	2	27.0332	1700000
APPSFADLGK	SC	36.58	1001.5182	10	6.2	501.76947	2	33.4927	41400
YGLRYF	SC	36.38	817.4122	6	0.6	409.71365	2	38.2383	428000
SPYYGYYGLRYF	SC	36.58	1547.7085	12	-1.2	774.8606	2	48.4606	2450000
YGYYGLRYF	SC	36.58	1200.5604	9	5.3	601.29065	2	45.8883	309000
GYGGFGKFH	SC	36.57	968.4504	9	0.4	485.2327	2	26.9859	184000
FGGGRGGFGGG	SC	36.58	924.4202	11	2.2	463.21838	2	21.3086	164000
RGGFGGGRGGFGG	SC	36.57	1137.5427	13	5	569.78149	2	20.13	171000
FGGGRGGFG	SC	28.31	810.3773	9	2.9	406.19708	2	22.1687	243000
YVLLPLFL	SC	21.9	976.5997	8	-6.6	489.30392	2	57.3207	/
SPQFGTHYW	SC	35.58	1121.493	9	1.1	561.75439	2	36.6033	187000
YYGLRYF	SC	36.57	980.4756	7	5.2	491.24762	2	42.7846	3660000
SSPYYGYYGLRYF	SC	36.56	1634.7405	13	-1.5	818.37634	2	48.4606	6920000
FGGGRGGFGG	SC	36.45	867.3987	10	1.3	434.70721	2	21.7742	201000
SGEVFKGFVFR	SC	36.58	1271.6663	11	2.2	636.8418	2	41.7405	415000
PYYGYYGLRYF	SC	36.58	1460.6765	11	0.3	731.34576	2	48.596	341000
APGIIPR	SC/SCOL	36.17	722.4439	7	0.1	362.22925	2	25.828	74700
AAPFG	SCOL	22.31	461.2274	5	2.2	462.23709	1	27.3804	20500
SPGPVPRPR	SCOL	37.76	961.5457	9	0.2	481.78168	2	15.7649	157000
NPGFDFTR	SCOL	40.37	952.4402	8	-1.5	477.22812	2	31.1949	253000
SPGPVPRP	SCOL	36.83	805.4446	8	-3.6	403.72934	2	26.6698	159000
PGPVPRPR	SCOL	40.58	874.5137	8	2.7	438.2666	2	20.669	238000
PGPVPRP	SCOL	38.43	718.4126	7	3.9	719.4248	1	26.4669	1780000
LSPGPVPRPRSKRL	SCOL	22.38	1558.9419	14	5.8	390.74619	4	19.0401	79600
AAPPP	SCOL	28.29	451.2431	5	6.4	452.25458	1	17.3246	43900
ARKFGPKGYGFGGGA	SCOL	27.37	1468.7574	15	4.3	490.59665	3	22.8708	133000
SPGPVPRPRSKR	SCOL	38.35	1332.7738	12	-0.3	445.26642	3	15.1878	124000

### 3.11 GO and KEGG enrichment analysis

**Figure 11:**
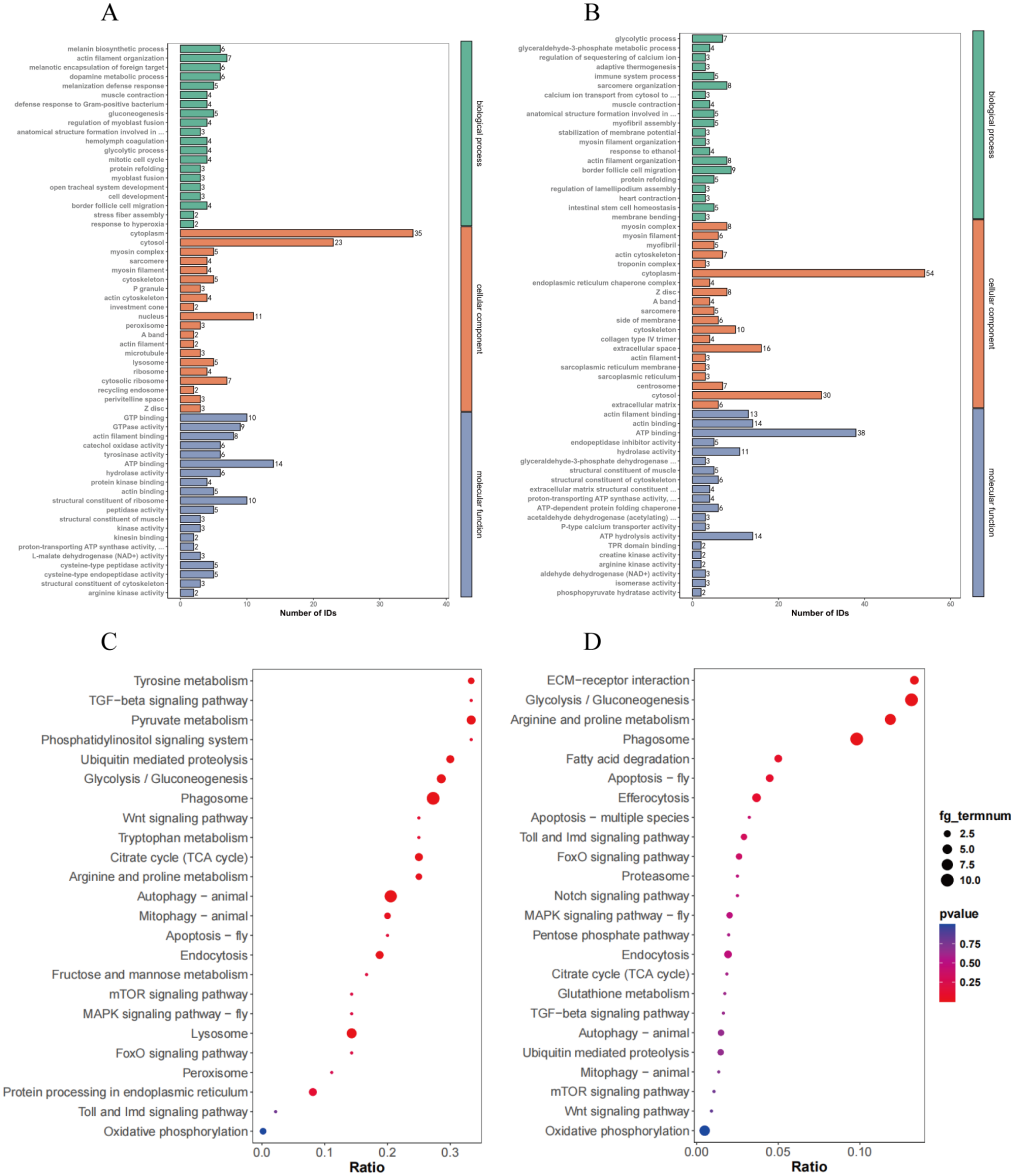
Enrichment analysis of proteins from scorpio and scolopendra. **(A, B)** GO functional enrichment analysis of scorpio **(A)** and scolopendra **(B)**, covering three dimensions: biological processes (BP), cellular components (CC), and molecular functions (MF). **(C, D)** The top 24 significantly enriched KEGG pathways for whole scorpio **(C)** and scolopendra **(D)**.

Finally, we performed GO functional and KEGG pathway enrichment analyses on the differentially expressed proteins identified from the scorpio and scolopendra groups. Among the 263 differentially expressed proteins identified in the scorpio group, 110 proteins had pathway information; among the 470 differentially expressed proteins identified in the scolopendra group, 171 proteins had pathway information. See the **Supplementary Tables S1**, **S2** for details. In the GO functional enrichment analysis, differentially expressed proteins from scorpio and scolopendra showed significant enrichment across all three dimensions—BP, CC, and MF—as illustrated in **Figures 11A, B**. Notably, in terms of GO functions, both groups were enriched in processes such as actin filament organization, defense response, and ATP binding. Species-specific enrichment patterns were also prominent. Specifically, scorpio differentially expressed proteins showed a significant enrichment in GO terms related to cysteine protease activity. In contrast, scolopendra differentially expressed proteins were specifically enriched in GO terms related to glycolytic metabolism and lysosomal function.

The KEGG pathway enrichment analysis presented the top 24 significantly enriched pathways in a bubble plot. There is a clear species-specific divergence in pathway enrichment patterns. Specifically, differentially expressed proteins from the scorpio were significantly enriched in regulatory pathways related to autophagy/mitophagy, Toll and Imd signaling, and TGF-β signaling (**Figure 11C**). In contrast, the unique pathways enriched in differentially expressed proteins from the scolopendra were concentrated in the efferocytosis pathway and proteasome pathways (**Figure 11D**).

## 4 Discussion

Diabetic wounds are a common type of hard-to-heal wound in clinical practice, characterized primarily by persistent inflammation and susceptibility to infection (23). Current treatment regimens still have significant limitations in terms of efficacy. During the development of these wounds, dysfunction of effector immune cells—such as macrophages—plays a key regulatory role in impairing wound healing (24–26). Jiedu Shengji Ointment is formulated according to the “sovereign, minister, assistant, and messenger” theory of herbal combination. It consists of scorpio, scolopendra, frankincense, and myrrh blended into a topical ointment. In this formula, scorpio and scolopendra serve as the sovereign herbs, while frankincense and myrrh are used together as the minister herbs. As a classic medicinal pair widely used in clinical TCM practice, scorpio and scolopendra have been extensively studied and confirmed to possess significant anti-inflammatory pharmacological activity. Research indicates that the scorpio-scolopendra combination can significantly inhibit the expression of NLRP3, Caspase-1, and IL-1β in airway epithelial cells. This blocks pyroptosis and effectively alleviating inflammatory conditions in mice (27). Frankincense is a commonly used Chinese herbal medicine for promoting blood circulation and relieving pain. Modern pharmacological studies have confirmed that frankincense exerts anti-inflammatory effects by targeting the NLRP3 inflammatory pathway through the active components in its essential oil (28). At the same time, frankincense also exerts anti-inflammatory effects through multiple mechanisms, including the inhibition of the NF-κB signaling pathway via its constituent boswellic acid (29). In traditional Chinese medicine, frankincense is often combined with myrrh to effectively treat traumatic injuries, inflammatory lesions, and chronic pain conditions. Myrrh is also a classic TCM herb used to promote blood circulation, relieve pain, reduce swelling, and promote tissue regeneration. It has a rich anti-inflammatory chemical basis. Modern pharmacological research has confirmed that myrrh, through its core active component gallic acid, mediates microglial polarization and alleviates inflammatory damage by regulating the NLRP3 signaling pathway (30). Additionally, recent studies have found that various unique natural compounds in myrrh resin also possess excellent anti-inflammatory activity. These findings further enrich the anti-inflammatory active substances and targets of myrrh (31). *Zongfutang Gongxuan Liangfang* is a highly representative surgical treatise from the Qing Dynasty. The book explicitly records that a topical ointment formulated by combining scorpio and scolopendra with frankincense and myrrh can be used to treat various surgical conditions. These include abscesses, boils, toxic sores, chronic non-healing wounds, and inflammatory swelling and pain. In summary, the combination of scorpio, scolopendra, frankincense, and myrrh can leverage their pharmacological and traditional therapeutic advantages in reducing inflammation and swelling while promoting wound repair. It offers promising prospects for wound healing applications. Therefore, this study aims to clarify the role of the Jiedu-Shengji Ointment in promoting the healing of diabetic wounds and to explore its potential molecular mechanisms.

This study used STZ to establish a mouse model of diabetes. Following intraperitoneal injection of STZ, blood glucose levels in the mice rose continuously and eventually stabilized at >16.7 mmol/L. During the maintenance phase, the diabetic mouse model successfully replicated the classic “three excesses and one deficiency” symptoms typical of advanced human diabetes (excessive thirst, excessive hunger, excessive urination, and weight loss) (32). Based on this, full-thickness skin defect wounds were created. Mice in the model group exhibited significantly reduced wound healing rates, decreased granulation tissue formation, and delayed epithelial migration. This indicates that the model effectively simulates the impaired wound healing observed in clinical diabetic patients. After 14 days of treatment with JDSJG (Jiedu-Shengji Ointment), the wound closure rate in STZ-induced diabetic mice significantly accelerated. It approached that of the normal wound group. Furthermore, JDSJG treatment significantly improved damage to organelles such as mitochondria and the endoplasmic reticulum, as well as the state of inflammatory cell infiltration in the wound tissue. It also reduced the release of TNF-α, IL-1β, and IL-18, while promoting the polarization of macrophages toward the M2 phenotype. These results suggest that JDSJG may exert its wound-healing effects by inhibiting the pyroptosis pathway, regulating the phenotypic polarization of macrophages, and improving the local immune microenvironment at the wound site. In this study, the marketed drug HAMDY was selected as a positive control to objectively evaluate the efficacy and clinical application potential of JDSJG. Based on the experimental results, JDSJG and HAMDY demonstrated comparable overall therapeutic effects. Both effectively promoting the healing of diabetic wounds. Although some parameters indicated a trend toward superior efficacy for JDSJG, this advantage was not supported by statistically significant results. Additionally, some parameters in this study exhibited wide error bars, reflecting a certain degree of variability in the data within each group. The reasons for this are twofold. First, there is inherent individual heterogeneity in blood glucose levels and the degree of metabolic dysfunction among the induced diabetic mice. Also, the wound repair microenvironment is difficult to standardize completely. Second, inflammatory factors such as IL-1β and IL-18, as well as NLRP3 pathway proteins, are highly sensitive to external stimuli and are inherently prone to fluctuations in expression. Despite these objective variations, the differences between groups were statistically significant. Furthermore, the trends in indicator changes aligned with experimental expectations. Therefore, these variations do not affect the reliability of the study’s conclusions and mechanistic analysis. In future studies, we will increase the number of biological replicates to mitigate experimental errors caused by individual differences. We will further validate the potential therapeutic benefits of JDSJG in promoting wound healing.

In this study, an *in vitro* pyroptosis-inflammation model was established using LPS-induced RAW264.7 macrophages. Macrophages within diabetic hard-to-heal wound sites generally exhibit persistent M1 polarization. They are accompanied by typical pathological features such as excessive activation of the NLRP3 inflammasome and massive secretion of pro-inflammatory factors (33, 34). LPS intervention can effectively activate the NLRP3 signaling pathway in macrophages, inducing M1 polarization and pyroptosis (35). The *in vitro* results of this study show that following LPS and nigericin treatment, RAW264.7 cells developed characteristic membrane pore structures. Also, the levels of NLRP3 gene and protein expression were significantly elevated. The cells exhibited a tendency toward M1 phenotypic polarization. Furthermore, the release of pro-inflammatory factors such as IL-1β and IL-18 in the cell culture supernatant increased markedly. These changes closely correspond to the pathological alterations observed in macrophages from diabetic wounds. Following treatment of LPS- and nigericin-induced RAW264.7 cells with JDSJG, LDH release decreased, cell morphology improved, and the formation of membrane pores was reduced. At the same time, secretion levels of TNF-α, IL-1β, and IL-18 were significantly lowered, This further confirms the direct inhibitory effect of JDSJG on macrophage pyroptosis. It is worth noting that this study used both Western blot and ELISA to detect IL-1β expression, and the results showed different trends. In the LPS+Nigericin stimulation group, both intracellular and supernatant IL-1β levels were higher than those in the LPS-only group. This is consistent with the classical mechanism by which Nigericin activates the NLRP3 inflammasome and promotes the cleavage, maturation, and release of pro-IL-1β. This demonstrates that this cellular model system is stable and reliable. Following JDSJG intervention, intracellular IL-1β (WB) in the LPS+Nigericin+JDSJG group showed a trend toward lower levels compared to the LPS+JDSJG group (no statistical difference). In contrast, secreted IL-1β in the supernatant (ELISA) was significantly higher than in the LPS+JDSJG group, with the difference being statistically significant. This discrepancy primarily stems from the differences in the detection targets of the two methods: Western blot reflects intracellular IL-1β protein levels, whereas ELISA detects mature IL-1β secreted into the cell supernatant. As a classic NLRP3 agonist, nigericin strongly promotes inflammasome-mediated protein cleavage and secretion. Concurrently, pyroptosis-induced increased cell membrane permeability accelerates the extracellular release of IL-1β. These factors ultimately lead to the observed discrepancy in the detection trends between intracellular proteins and secreted proteins in the cell culture supernatant.

A growing body of evidence suggests that activation of the NLRP3/Caspase-1/GSDMD signaling pathway may influence the function of immune cells in the wound microenvironment by regulating pyroptosis and inflammatory responses (10). The NLRP3/Caspase-1/GSDMD pathway is stimulated by high glucose, LPS, damage-associated molecular patterns (DAMPs), ROS, and other factors to facilitate inflammasome assembly and activation, as well as GSDMD-N-mediated pore formation (7–9). Once membrane pores form, they disrupt cellular osmotic balance, triggering ion influx, cell swelling and lysis, and the release of intracellular contents, ultimately leading to programmed pyroptosis (36, 37). On the other hand, it promotes the massive release of pro-inflammatory factors such as IL-1β and IL-18 into the extracellular wound microenvironment. This induces the infiltration and aggregation of peripheral immune cells at the wound site, continuously amplifying the local inflammatory response. As a result, a vicious cycle of chronic inflammation is created, which impedes the wound healing process (38). Abnormal activation of this pathway has also been documented in various chronic inflammatory diseases. This confirms the potential link between pyroptosis and impaired tissue repair (39, 40). Previous studies have demonstrated that in diabetic wound models, excessive activation of the NLRP3 inflammasome drives macrophage pyroptosis, leading to the sustained release of pro-inflammatory factors such as IL-1β and IL-18, thereby exacerbating the inflammatory microenvironment at the wound site (41). Teh et al. also systematically summarized the potential of the NLRP3 inflammasome pathway as a therapeutic target for diabetic wound inflammation. They noted that targeting these pathways can effectively improve wound healing (8). This view is consistent with the findings of the present study. Furthermore, we observed a significant downregulation of pyroptosis-related proteins (such as NLRP3, ASC, Caspase-1, and GSDMD) in wound tissue following JDSJG treatment. *In vitro* experiments showed that the NLRP3 agonist nigericin further enhanced LPS-induced pyroptosis and M1 polarization in macrophages, whereas JDSJG intervention effectively attenuated these effects. At the molecular level, crosstalk between macrophages and fibroblasts may play a key role in this process. In a radiation-induced myocardial injury model, cardiac fibroblasts co-cultured with post-irradiation macrophages exhibited a dose-dependent increase in the fibrosis markers α-SMA and Collagen I. However, blocking NLRP3-mediated pyroptosis significantly reduced the expression of these fibrosis markers in the co-cultured fibroblasts (42). This suggests that JDSJG may downregulate pro-inflammatory cytokine secretion by modulating the NLRP3/Caspase-1/GSDMD signaling pathway. By doing so, it lifts the functional inhibition of fibroblasts and creating favorable conditions for extracellular matrix repair.

Current research indicates that the pharmacological effects of scorpio and scolopendra primarily depend on the bioactive peptides they contain (43–45). To further elucidate the molecular basis of these effects, this study employed proteomics techniques for in-depth analysis. This study identified 472 and 1,395 peptides in scorpio and scolopendra, respectively, with 25 peptides common to both. GO functional enrichment analysis revealed that the differentially expressed proteins identified in the whole scorpio and scolopendra groups showed significant enrichment across three dimensions: biological processes, cellular components, and molecular functions. This suggests functional diversity in their protein composition. Notably, the differentially expressed proteins from scorpio and scolopendra share both commonalities and differences in GO functions. KEGG pathway enrichment analysis further revealed these distinctions. Although neither group showed direct enrichment in the classical pyroptosis pathway, the differentially expressed proteins from scorpio were significantly enriched in autophagy/mitochondrial autophagy, Toll/Imd signaling, and TGF-β signaling pathways. Existing studies indicate that autophagy exerts anti-inflammatory effects by clearing damaged mitochondria and inhibiting NLRP3 inflammasome activation (46, 47); the Toll signaling pathway is a key regulatory pathway of the innate immune response, and its activation can modulate the expression of inflammatory factors (48); while the TGF-β signaling pathway is closely associated with the polarization of macrophages toward the M2 phenotype and participates in inflammation resolution and tissue repair (49). These results suggest that scorpio extracts may be involved in NLRP3 inflammasome activation and M2 macrophage polarization. In contrast, the differentially expressed proteins in scolopendra extract are uniquely enriched in phagocytosis and the proteasome pathway. Phagocytosis is a key process for clearing cellular debris. Its effective execution prevents persistent inflammation and tissue damage; the proteasome pathway is responsible for degrading misfolded or damaged proteins, regulating cellular homeostasis and inflammatory signal transduction. This suggests that scolopendra extracts may exert their effects by promoting the clearance of pyroptotic fragments and regulating protein degradation. In summary, scorpio and scolopendra extracts may play complementary roles in promoting tissue repair. This finding provides new insights into the anti-inflammatory and macrophage function-regulating properties of JDSJG. It also offers new evidence for its clinical application in diabetic wounds.

This study still has certain shortcomings and limitations. First, the current results indicate that JDSJG promotes diabetic wound healing, reduces inflammatory mediators, and lowers the expression of proteins associated with the NLRP3/Caspase-1/GSDMD pathway. However, this study primarily employed agonist activation and phenotypic detection strategies. Reverse genetics validation using pyroptosis-specific inhibitors has not yet been conducted. Future studies will employ gene silencing/knockout and inhibitor blockade experiments to confirm these findings. Second, this study did not include a control group using a simple ointment base (placebo excipient), making it impossible to rule out the potential influence of the ointment base itself (e.g., petroleum jelly) on diabetic wound repair. Additionally, systematic testing of the ointment’s stability during the treatment period—such as the uniformity of active ingredients in the petroleum jelly base—was not conducted. Future studies will include a blank base control group and refine stability-related tests to further advance the standardized development of this topical compound formulation. Third, although the LPS-induced macrophage model shares similarities with the inflammatory phenotype of macrophages in diabetic wounds, limitations remain: ①*in vitro* single-cell models lack the multicellular interactions and extracellular matrix microenvironment found in wounds;②acute LPS modeling cannot simulate chronic pathological conditions associated with diabetes, such as systemic hyperglycemia and microcirculatory abnormalities. Future studies should employ co-culture systems, animal models, and various cellular models to conduct multi-level validation. Finally, traditional Chinese medicine formulas are characterized by their multi-component and multi-target nature. This study focused on investigating the active components and mechanisms of action of the two herbs, whole scorpio and scolopendra. The active constituents of frankincense and myrrh, as well as their mechanisms of action in regulating macrophages, remain unclear. Related research has been included in our future research plans.

## Data Availability

The datasets presented in this study can be found in online repositories. The names of the repository/repositories and accession number(s) can be found in the article/**Supplementary Material**.
